# Expression of the SIRT2 Gene and Its Relationship with Body Size Traits in Qinchuan Cattle (*Bos taurus*)

**DOI:** 10.3390/ijms16022458

**Published:** 2015-01-22

**Authors:** Lin-Sheng Gui, Ya-Ran Zhang, Gui-Yao Liu, Lin-Sen Zan

**Affiliations:** 1 College of Animal Science and Technology, Northwest A&F University, Yangling 712100, China; E-Mails: guilinsheng1234@163.com (L.-S.G.); zhang_ya_ran@126.com (Y.-R.Z.); 2 Institute of Soil and Water Conservation, Chinese Academy of Science and Ministry of Water Resource, Yangling 712100, China; E-Mail: lgyyuanyi2008@163.com; 3 National Beef Cattle Improvement Center of Northwest A&F University, Yangling 712100, China

**Keywords:** SIRT2 gene, expression study, body size traits, Qinchuan cattle

## Abstract

Silent information regulator 2 (SIRT2) is a member of the sirtuin family of class III NAD (nicotinamide adenine dinucleotide)-dependent protein deacetylases and may regulate senescence, metabolism and apoptosis. The aims of this study were to investigate whether the SIRT2 gene could be used as a candidate gene in the breeding of Qinchuan cattle. Real-time polymerase chain reaction (RT-PCR) results showed that among all types of tissue that were analyzed, the highest mRNA expression levels of the gene were found in subcutaneous fat. DNA sequencing of 468 individual Qinchuan cattle identified two novel, single nucleotide polymorphisms (g.19501 C > T and g.19518 C > T) in the 3' untranslated region (3'UTR) of the SIRT2 gene. The frequencies of SNP g.19501 C > T and g.19518 C > T were in Hardy-Weinberg disequilibrium in all the samples (chi-square test, *χ*^2^ < *χ*_0.05_^2^). An association analysis showed that the two loci were significantly correlated with some body size traits and the H_2_H_2_ (-CT-CT-) diplotypes performed better than other combinations. These results indicated that the variations in the SIRT2 gene and their corresponding genotypes may be considered as molecular markers for economic traits in cattle breeding.

## 1. Introduction

In mammals, the yeast silent information regulator factor 2 (SIR2) are called sirtuins; they belong to the family of class III nicotinamide adenine dinucleotide (NAD)-dependent histone deacetylase [1]. Seven members of the sirtuin family have been identified to date, and designated as SIRT1-SIRT7. They share a conserved central deacetylase domain but have different *N*- and *C*-termini. These proteins play complex and important roles in aging-related pathological conditions, such as cancer and the deregulation of metabolism [2]. Among them, SIRT2 is a cytoplasmic protein that may be either beneficial or detrimental for cell survival, depending on the conditions [3]. Studies of the central nervous system of mice have suggested that the SIRT2 gene is ubiquitously expressed as NAD^+^-dependent protein deacetylases [4] and it appears to be involved in mediating genomic silencing, DNA repair, and longevity [5,6].

SIRT2 is a NAD^+^-dependent protein deacetylase of several substrate proteins in the cytoplasm. Particularly, SIRT2 has been implicated in adipocyte differentiation through modulation of acetylation/phosphorylation of forkhead box O1 (FOXO1) [7]. Overexpression of SIRT2 in 3T3-L1 cells causes FoxO1 binding to the promoter of peroxisome proliferator-activated receptor-gamma (PPARγ) and subsequently represses its activity, which leads to abnormal mitochondrial morphology and inhibits adipogenesis [8]. Transcriptional repression of SIRT2 results in an inhibition of fatty acid oxidation (FAO) and energetic uncoupling via deacetylate hypoxia-inducible factor 1α (HIF1α) *in vivo* and *in vitro* [9]. Cell-based studies have suggested that SIRT2 deacetylates and stabilizes phosphoenolpyruvate carboxykinase1 (PEPCK1), thereby modulating the cellular response to glucose [10]. Combined, all of these findings support the hypothesis that SIRT2 is a potential candidate gene for the selection of growth-related traits in livestock.

Few SIRT2 variants have been reported to date for cattle, as most research has focused on rodents and human models [11]. The Qinchuan breed of cattle (*Bos taurus*) used in our research is one of the outstanding indigenous breeds in China, known for its excellent adaptability and physical features. However, it has obvious weaknesses compared to imported commercial beef cattle breeds, such as slow growth and underdeveloped hind hip [12]. Therefore, this study aimed to predict SIRT2 gene function in this cattle breed using bioinformatics information, in order to analyze tissue expression pattern using Real-time PCR, and to identify associated quantitative traits for the benefit of cattle breeding and genetics.

## 2. Results and Discussion

### 2.1. Molecular Cloning and Sequence of the Bovine SIRT2 Gene

Based on sequencing of a PCR product that encodes the SIRT2 cDNA of Qinchuan cattle, comparison of the SIRT2 amino acid sequence that we obtained with seven other animal species from GenBank revealed the following similarities (Table 1). *Sus scrofa* (93.30%), *Homo sapiens* (88.92%), *Rattus norvegicus* (88.00%), *Mus musculus* (85.27%), *Gallus gallus* (68.63%), *Danio rerio* (61.93%) and *Drosophila melanogaster* (49.86%). The relatively high amino acid similarity observed among mammalians (85.27%–93.30%) suggested that the SIRT2 gene was more conserved within this group.

**Table 1:** Comparative analysis of SIRT2 gene amino acid sequence of different animals.

Species	GenBank Accession	Similarity
*Sus scrofa*	NP_001107743.1	93.30%
*Homo sapiens*	NP_036369.2	88.92%
*Mus musculus*	NP_001116237.1	85.27%
*Rattus norvegicus*	NP_001008369.1	88.00%
*Gallus gallus*	NP_001088636.1	68.63%
*Drosophila melanogaster*	NP_001287422.1	49.86%
*Danio rerio*	NP_955890.1	61.93%

**Figure 1:**
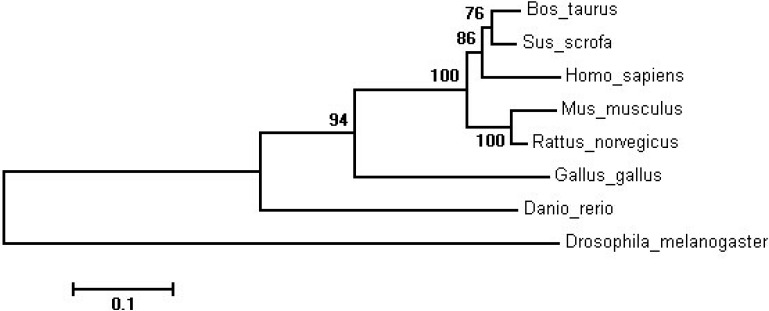
Phylogenetic tree of the SIRT2 gene in different species.

To better understand the relationship between bovine SIRT2 and the potential evolutional process, we constructed the phylogenetic tree based on amino acid sequence of SIRT2 (Figure 1). It showed that the bovine SIRT2 is phylogenetically closest to pig SIRT2 and then to human, with the mouse and rat forming a separate group, while the non-mammalian species formed an even more distant group.

### 2.2. Ontogenic Expression of SIRT2 in Qinchuan Cattle

**Figure 2:**
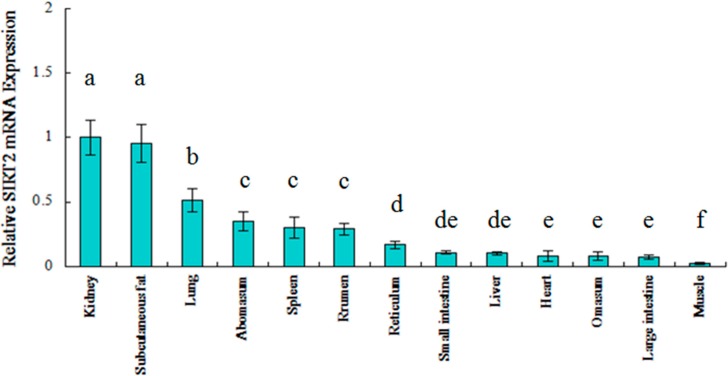
Tissue expression analysis of SIRT2 mRNA in Qinchuan cattle. Different lowercase letters above the bars (a–f) indicate significant difference between tissues (*p* < 0.05).

Tissue distribution analysis in pigs has indicated that sirtuin genes are expressed ubiquitously and with the highest abundance in brain, spinal cord and genital tissue [13]. In a calorie-restriction model for rats, SIRT2 was expressed predominantly in white adipose and kidney tissue [14]. Human SIRT2 is expressed in a variety of tissues, with high levels of expression in skeletal muscle and brain tissue [15]. To date, there have been no studies of the expression pattern of bovine SIRT2. We performed RT-PCR to determine the expression of SIRT2 in different tissues. The relative expression results were obtained using the 2^−ΔΔ*C*t^ method, which was first normalized to the geometric mean of β-actin, RPS9 and GAPDH. As shown in Figure 2, SIRT2 was widely expressed in Qinchuan cattle. Moreover, the comparison of SIRT2 gene expression in diverse tissues demonstrated that the bovine SIRT2 gene was highly expressed in kidney, subcutaneous fat, and lung tissue, moderately expressed in rumen, spleen and abomasum tissue, and only slightly expressed in muscle, large and small intestine, heart, omasum, liver and reticulum tissue. There seem to be clear connections (directly or indirectly) between body size traits and modulation by SIRT2 genes in Qinchuan cattle, which fits to what we know about the important functions of SIRT2 genes in metabolism, especially in gluconeogenesis and lipid oxidation [16].

### 2.3. Genetic Polymorphism of Qinchuan Cattle SIRT2 and χ^2^ Test

**Figure 3:**
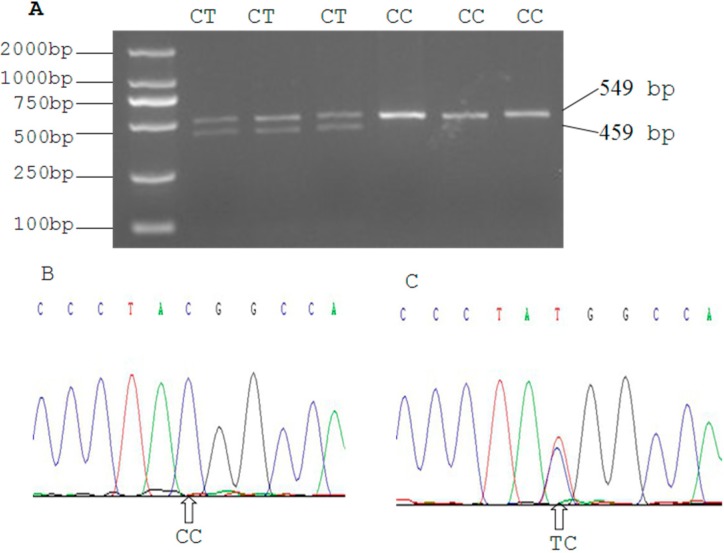
(**A**) PCR-RFLP detection results of SIRT2 gene PCR product (19,501 bp locus); (**B**,**C**) The sequencing maps of the novel SNP of SIRT2 gene (19,501 bp locus).

**Figure 4:**
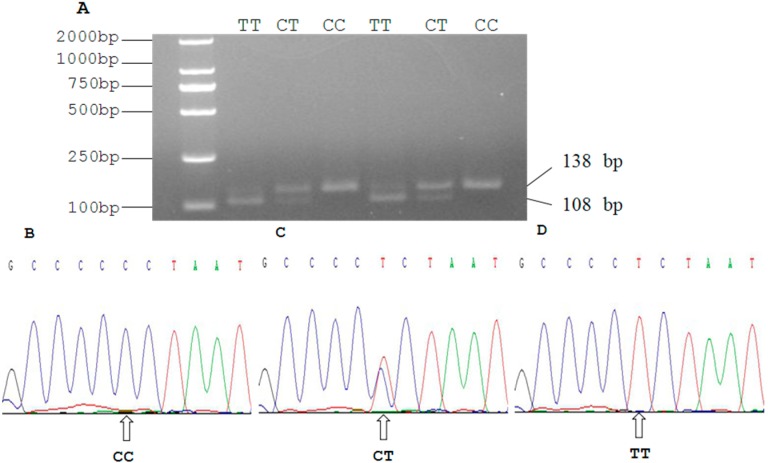
(**A**) PCR-RFLP detection results of SIRT2 gene PCR product (19,518 bp locus); (**B**–**D**) The sequencing maps of the novel SNP of SIRT2 gene (19,518 bp locus).

Sequence analysis of the SIRT2 gene revealed two C > T mutations in the 3'UTR region at 19,501 bp (Figure 3) and 19,518 bp (Figure 4). At the g.19501 C > T locus digestion of the 549 bp PCR fragment of SIRT2 3'UTR with BstX1 resulted in fragment lengths of 549, 459, and 90 bp for genotype CT, and 549 bp for genotype CC. The frequency of allele C was dominant in Qinchuan cattle, and genotype CC was more frequent than CT. At the g.19518 C > T locus, digestion of the 138 bp PCR fragment of SIRT2 3'UTR with Xba1 resulted in fragment lengths of 138 bp for genotype CC; 138, 108, and 30 bp for CT, and 108, 30 bp for TT. The frequency of allele C was dominant in Qinchuan cattle and genotype CT was more frequent than other genotypes.

We found that the g.19501 C > T locus had 2 genotypes, and the genotype TT was not observed in the sampled animals. The absence of that genotype in this population of Qinchuan cattle might mean that it does not exist in the population, or that the size of the experimental population was too small to capture its full genetic variation.

Based on analysis of genotype and allele frequencies (Table 2), we found that for the g.19501 C > T mutation, the CT genotype (20.09%) was less frequent than the wild allele CC (79.91%). Allele frequencies, gene heterozygosity (He), effective allele numbers (Ne) and polymorphism information content (PIC) at the current locus were 0.8996 (C), 0.1004 (T), 0.1807, 1.2205 and 0.1644, respectively. For the g.19518 C > T mutation, the CT genotype was the most prevalent (43.16%) followed by CC (41.03%) and TT (15.81%). The values of He, Ne, and PIC at the current locus were 0.6261 (C), 0.3739 (T), 0.4682, 1.8805 and 0.3586, respectively.

**Table 2:** Genotype frequencies (%) of the SIRT2 gene for the SNPs in the Qinchuan cattle populations. Note: HWE, Hardy-Weinberg equilibrium; χ_0.05_^2^ = 5.991, χ_0.01_^2^ = 9.21.

Locus	Genotypic Frequencies (N)			Total	Allelic Frequencies		χ^2^ (HWE)	PIC	He	Ne
	CC	CT	TT		C	T				
g.19501 C > T	0.7991	0.2009	0	468	0.8996	0.1004	5.8328	0.1644	0.1807	1.2205
g.19518 C > T	0.4103	0.4316	0.1581	468	0.6261	0.3739	2.8581	0.3586	0.4682	1.8805

According to the conventions for PIC classification (PIC value <0.25 is considered low polymorphism, 0.25–0.50 is intermediate polymorphism, and >0.50 is high polymorphism), our data showed that g.19501 C > T had an intermediate level of polymorphism, while g.19518 C > T locus had low polymorphism. The genotypic distributions of these two mutations were in Hardy-Weinberg disequilibrium (chi-square test, *χ*^2^ < *χ*_0.05_^2^), indicating that the genotypic frequencies had been affected by selection, mutation or migration.

### 2.4. Effects of Single Marker on Body Size Traits

Analysis of the SIRT2 gene in five main breeds of Chinese cattle demonstrated that the polymorphism of g.4140A > G was significantly related to body weight in Nanyang cattle [17]. Here, two novel SNPs (g.19501 C > T and g.19518 C > T) were found in the SIRT2 gene. Association results of single markers with nine economic traits in the Qinchuan population are shown in Table 3. The g.19501 C > T was significantly associated with body length. Individuals with genotype CC had significantly greater body length than those with genotype CT (*p* = 0.0022). For g.19518 C > T, individuals with genotype TT had significantly greater rump length compared with genotype CC (*p* = 0.0025), indicating that allele T might be associated with an increase in rump length in Qinchuan cattle. Such associations remained significant even after Bonferroni correction (*p* < 2.78 × 10^−3^).

**Table 3:** Association of different genotypes of single nucleotide polymorphisms (SNPs) in SIRT2 with body size traits in *Qinchuan* cattle. Note: Values are shown as the least squares means ± standard error. ^a,b^ Means with different superscripts are significantly different (*p* < 2.78 × 10^−3^) after Bonferroni correction. Body length (BL), withers height (WH), hip height (HH), rump length (RL), hip width (HW), chest depth (CD), chest circumference (CC), and pin bone width (PBW), fat thickness (BF) and ultrasound loin muscle area (ULA).

Locus	Genotypes	BL (cm)	WH (cm)	RL (cm)	HW (cm)	CD (cm)	CC (cm)	PBW (cm)	BF (cm)	ULA (cm^2^)
g.19501 C > T	CC (374)	132.608 ± 0.478 ^a^	119.222 ± 0.481	41.664 ± 0.201	38.286 ± 0.258	58.345 ± 0.315	161.993 ± 0.734	18.409 ± 0.142	0.873 ± 0.014	45.188 ± 0.673
	CT (94)	129.210 ± 0.721 ^b^	117.329 ± 0.770	41.287 ± 0.351	36.968 ± 0.324	57.159 ± 0.447	158.596 ± 0.959	17.776 ± 0283	0.860 ± 0.023	42.343 ± 0.962
	*P*-value	0.0022	0.0593	0.3071	0.0204	0.1051	0.0193	0.0387	0.3685	0.0367
g.19518 C > T	CC (192)	130.844 ± 0.672	118.612 ± 0.671	41.099 ± 0.278 ^b^	37.177 ± 0.359	57.419 ± 0.440	159.276 ± 1.023	17.849 ± 0.197	0.850 ± 0.019	44.600 ± 2.162
	CT (202)	132.810 ± 0.655	118.381 ± 0.654	41.683 ± 0.271	38.649 ± 0.350	58.369 ± 0.429	162.552 ± 0.998	18.629 ± 0.193	0.883 ± 0.021	45.017 ± 2.246
	TT (74)	133.682 ± 1.082	120.696 ± 0.882	42.608 ± 0.447 ^a^	38.500 ± 0.578	59.176 ± 0.522	163.203 ± 1.648	18.459 ± 0.318	0.890 ± 0.031	43.566 ± 1.554
	*P*-value	0.0175	0.1004	0.0025	0.0031	0.0267	0.0127	0.0380	0.2007	0.2068

Sequence alignment demonstrated that these two SNPs located in the 3'UTR did not change the structure of their encoded proteins. However, recent research has provided evidence that mutation in the 3'UTR could affect protein expression and phenotype by altering the stability of mRNA [18]. In Texel sheep, Clop *et al.* (2006) [19] reported a significant association between a G/A polymorphism in the 3'UTR of the MSTN gene and the phenotype of muscular hypertrophy. Ren *et al.* (2010) [20] identified a *HeaⅢ* polymorphism in the 3'UTR of the LHX4 gene that was associated with body mass and length in Nanyang cattle. Therefore, we hypothesized that 3'UTR mutation g.19501C > T and g.19518 C > T of SIRT2 may play significant roles in modifying gene expression patterns, which would therefore affect body size traits in cattle.

### 2.5. Linkage Disequilibrium (LD) and Haplotype Analysis

In regression analysis, *r*^2^ values above 0.33 might imply LD that is strong enough to be used for mapping [21]. In our study, the pair-wise D' and *r*^2^ values of the SNPs were 0.918 and 0.158, respectively. The *r*^2^ values in SIRT2 were lower than 0.33, indicating that those SNPs had little LD. The rate of recombination may therefore be high, and LD would hence be low in genovariation-dense regions [22].

In order to perform haplotype-based association analysis, three different haplotypes were constructed in SIRT2. The three most frequent haplotypes had a summed probability of 0.995, and those of frequency less than 0.05 were ignored (Table 4). Hap_1_ (-CC-) had the highest haplotype frequencies (62.10%), followed by Hap_2_ (-CT-), and Hap_3_ (-TT-). The haplotypes with high frequency have probably been present in the population for a long time, which may be directly or indirectly regulated by different rearing environments [23].

**Table 4:** Haplotypes of SIRT2 gene and their frequencies in Qinchuan cattle.

Haplotype	g.19501 C > T	g.19518 C > T	Frequency
Hap1	C	C	0.621
Hap2	C	T	0.279
Hap3	T	T	0.095
Hap4	T	C	0.005

### 2.6. Effects of Haplotype Combinations on Body Size Traits

We inferred that the effects of variation of a gene could be demonstrated more readily by integrating analysis of the haplotype combinations with the single locus effects. Then the effects of the combinations of the two SNPs were evaluated, and a total of five haplotype combinations were identified for further analysis. Compared with the combination results, individuals with H_2_H_2_ diplotypes performed better in terms of their body traits (Table 5). Specifically, the H_2_H_2_ diplotypes had significantly greater body length (*p* = 0.0004), withers height (*p* = 0.0005), hip width (*p* = 0.0024), chest depth (*p* = 0.0023) and pin bone width (*p* = 0.0019), than H_1_H_3_ diplotypes; such associations remained significant even after Bonferroni correction for multiple testing (*p* < 2.78 × 10^−3^). Based on our findings, we infer that the H_2_H_2_ diplotypes could be used as a molecular marker of combined genotypes for future selection of body size traits in Qinchuan cattle.

Several studies have reported that marker-assisted selection (MAS), which selects particularly for beneficial traits that have low heritability, can accelerate genetic gains dramatically compared to conventional breeding [24]. The use of MAS technology has demonstrated that many genes are related to growth [25], production [26] and meat quality traits [27] in livestock. Our previous study revealed significant associations between polymorphisms in SIRT1 and body size in Qinchuan cattle [28]. Functional studies of SIRT1 and SIRT2 showed that they share a conserved central deacetylase domain [29] and that both proteins inhibit proliferation and differentiation in adipocytes [8,30]. Those similar genetic effects can be attributed to their similar function in metabolism. These findings suggest that the SIRT2 gene may have an important influence on animal body size traits, thus making it useful in MAS in cattle.

**Table 5:** Associations of haplotypes with body size traits in Qinchuan cattle. Note: Values are shown as the least squares means ± standard error. ^a–c^ Means with different superscripts are significantly different (*p* < 2.78 × 10^−3^) after Bonferroni correction. Body length (BL), withers height (WH), hip height (HH), rump length (RL), hip width (HW), chest depth (CD), chest circumference (CC), and pin bone width (PBW), fat thickness (BF) and ultrasound loin muscle area (ULA).

Combined Genotypes	Body Measurement							Meat Quality Trait	
	BL (cm)	WH (cm)	RL (cm)	HW (cm)	CD (cm)	CC (cm)	PBW (cm)	BF (cm)	ULA (cm^2^)
Hap1/1 (188)	131.016 ± 0.658 ^b^	118.332 ± 0.674	41.309 ± 0.281	37.319 ± 0.357	57.582 ± 0.440	159.297 ± 1.016	17.899 ± 0.197 ^b^	0.851 ± 0.020	44.054 ± 1.152
Hap1/2 (168)	133.717 ± 0.696 ^a,b^	119.884 ± 0.713 ^a^	41.851 ± 0.297	39.089 ± 0.377 ^a^	58.919 ± 0.465	164.354 ± 1.075 ^a^	18.863 ± 0.209	0.891 ± 0.021	46.341 ± 1.073
Hap1/3 (34)	125.359 ± 1.520 ^c^	113.779 ± 1.584 ^b^	40.294 ± 0.660	35.735 ± 0.839 ^b^	55.588 ± 0.881 ^b^	153.970 ± 2.389 ^b^	17.088 ± 0.464 ^b^	0.811 ± 0.046	40.579 ± 1.741
Hap2/2 (18)	138.868 ± 1.776 ^a^	122.333 ± 1.399 ^a^	43.667 ± 0.907	40.726 ± 0.559 ^a^	60.944 ± 0.824 ^a^	168.121 ± 2.283 ^a^	19.500 ± 0.638 ^a^	0.927 ± 0.063	46.266 ± 1.271
Hap2/3 (56)	132.009 ± 1.204 ^a,b^	119.438 ± 1.234	41.857 ± 0.514	37.788 ± 0.653	58.161 ± 0.806	161.464 ± 1.861	18.161 ± 0.362	0.898 ± 0.036	43.347 ± 2.271
*P*-value	0.0004	0.0005	0.0028	0.0024	0.0023	0.0084	0.0019	0.1142	0.0192

## 3. Experimental Section

### 3.1. Bioinformatic Study

Sequence similarity between bovine SIRT2 protein and its homologue were obtained from Genbank using a search of the BLAST protein databases (http://blast.ncbi.nlm.nih.gov/Blast.cgi). Phylogenetic and molecular evolutionary analysis was conducted using ClustalX software, and the results were further analysed with Mega5.1 software. Numbers at each branch indicated the percentage of times a node was supported in 1000 bootstrap pseudoreplications, based on neighbor joining.

### 3.2. Collection of RNA Samples

Three purebred two-year-old bulls of the Qinchuan breed were purchased from the Experiment farm of the National Beef Cattle Improvement Center (Yangling, China). The animals were euthanized with a captive bolt gun and then exsanguinated. Samples were collected from 13 types of tissue: Heart, liver, spleen, lung, kidney, muscle, subcutaneous fat, rumen, reticulum, omasum, abomasum, and small and large intestine. They were frozen immediately in liquid nitrogen, and kept at −80 °C until analysis.

### 3.3. RNA Purification and cDNA Synthesis

Total RNA was isolated from each tissue sample using an RNA simple Total RNA kit (Tiangen, Beijing, China) according to the manufacturer’s instructions. The RNA was quantified using the NanoDrop 1000 spectrophotometer (Thermo Fisher Scientific Inc., Wilmington, DE, USA) at 260 nm. The ratios of OD260 and OD280 for all RNA isolates ranged from 1.8 to 2.0. The integrity of the RNA samples was verified by electrophoresis in a 0.8% agarose gel stained with 0.5 μg/mL ethidium bromide. Then a reverse transcription reaction was performed for the first-strand cDNA synthesis (TaKaRa, Dalian, China). Firstly, to destabilize the secondary structure of RNA, total RNA for each individual was incubated at 65 °C for 5 min; secondly, 10 μL of total RNA was combined with 5× PrimeScript Buffer, 0.5 μL RNase Inhibitor, 0.5 μL PrimeScript RTase, and RNase water, with a total volume 20 μL. The mixture was put on a thermal cycler, and the first-strand cDNA synthesis was accomplished using the program of 37 °C 10 min, 42 °C for 30 min, and for 70 °C 10 min.

### 3.4. SYBR Green RT-PCR Analysis of Expression Patterns

For normalization of the expression analyses, three reference genes were evaluated: β-actin (AY141970.1), RPS9 (NM_001101152.2), and GAPDH (NM_001034034). Each sample for Real-time PCR was conducted in triplicate and SIRT2 levels were quantified relative to the geometric mean of β-actin, RPS9 and GAPDH via the 2^−ΔΔ*C*t^ method. Primers were designed for the SIRT2 gene and reference genes (Table 6), and gene expression profiles were than analyzed by Real-time PCR using the SYBR Premix Ex TaqII (TaKaRa, Dalian, China). The program consisted of a pre-incubation cycle at 95 °C for 30 s, 40 quantification cycles of a 5 s denaturation at 95 °C, and 45 s annealing at 60 °C. PCRs were conducted in triplicate and then SIRT2 levels were then quantitated using 7500 System SDS Software V 1.4.0 (Applied Biosystems, Waltham, MA, USA).

**Table 6:** Primers used in these experiments.

Name	Function	Primer Sequence (5' to 3')	Tm (°C)	Product Length	Amplified Region
SIRT2	RT-PCR	CAACCTGGAGAAATACCGTCTT	61.0	166 bp	400–565
		CAGTCCTTTTTCCTTCAGCAG			
β-actin	Reference	CACCAACTGGGACGACAT	61.0	202 bp	320–521
		ATACAGGGACAGCACAGC			
RPS9	Reference	CCTCGACCAAGAGCTGAAG	61.0	64 bp	128–191
		CCTCCAGACCTCACGTTTGTTC			
GAPDH	Reference	CCAACGTGTCTGTTGTGGAT	61.0	80 bp	778–857
		CTGCTTCACCACCTTCTTGA			
Primer A	3'UTR region amplification	ACCCCTGACCTCACCAAAT	56.5	549 bp	19411–19959
		GCACCTTTCAGCACTCTTC			
Primer B	3'UTR region amplification	ACCCCTGACCTCACCAAAT	62.3	138 bp	19411–19548
		CCTGTGGCCCCCTGAGCAGTTAGAGTCTAG			

### 3.5. Animal Source, Data Collection and Genomic DNA Isolation

In total, 468 adult cows (that were female, 18–24 months old, and unrelated for at least three generations) were randomly selected from National Beef Cattle Improvement Center’s experiment farm (Yangling, China). Body measurement traits were measured as described previously [31], including body length, withers height, rump length, chest depth, hip width, chest circumference and pin bone width. Ultrasound was used to measure some additional traits on carcasses, namely fat thickness and ultrasound loin muscle area.

DNA was extracted from blood samples collected from the jugular vein prior to euthanasia and stored at 80 °C according to the standard phenol chloroform protocol [32]. DNA content was estimated by spectrophotometer. Genomic DNA was diluted to 50 ng/μL. All DNA samples were stored at 20 °C until subsequent analysis.

### 3.6. PCR Amplification and Sequencing

Based upon the sequence of the bovine SIRT2 gene (GenBank accession No. NM_001113531.1), two pairs of primers were designed to amplify it with PCR using primer premier 5 (Table 6). They produced two sequences, of 549 bp (Primer A) and 138 bp (Primer B) respectively. The PCR amplification product was a mixture of 20 μL composed of 50 ng DNA, 10 pM of each primer, 0.2 mM dNTP, 2.5 mM MgCl_2_ and 0.5 U Taq DNA polymerase (TaKaRa, Dalian, China). The cycling protocol consisted of denaturation for 5 min at 95 °C, 35 cycles of 94 °C for 30 s, annealing for 30 s, primer extension at 72 °C for 30 s, and a final extension performed at 72 °C for 10 min.

### 3.7. DNA Pooling, PCR-RFLP and DNA Sequencing

Thirty random individual DNA samples were mixed to form a DNA pool that was used to detect mutations in the SIRT2 gene. DNA sequencing was then applied to screen variations within the amplified regions of the DNA pool, and the products amplified from genomic DNA were imported directly into the BioXM software version 2.6.

Two SNPs (g.19501 C> T and g.19518 C > T) were identified within the 3'UTR of the SIRT2 gene based on DNA sequencing. Further analysis with Primer Premier 5.0 software revealed that the g.19501 C > T and g.19518 C > T were located in the *BstX* and *Xba* restriction sites, respectively. Therefore, genotyping of the two SNPs was performed using polymerase chain reaction-restriction fragment length polymorphism (PCR-RFLP). Aliquots of 10 μL of PCR products were digested with 10 U *BstX* (g.19501 C > T) and *Xba* (g.19518 C > T) for 8 h at 37 °C, respectively. The digested products were detected by electrophoresis on a 2.5% agarose gel stained with ethidium bromide. Finally, PCR products with different electrophoresis patterns were sequenced from both directions using the DNA sequencer (Applied Biosystems 3730 Genetic Analyzer, USA), and the results were analyzed by DNAMAN software version 5.2.2 (Madison, WI, USA).

### 3.8. Statistical Analysis

Several parameters including allelic frequencies, genotypic frequencies, He, Ne, Hardy-Weinberg equilibriums and PIC were analyzed statistically according to Nei’s methods [33,34]. LD and haplotype distributions of the SNPs were analyzed using the expectation maximization algorithm with Haploview software [35].

The association between single SNPs and body size traits were analyzed using the general linear models (GLM) procedure in SPSS (version 13.0). The linear model was used:

Y_ijklm_ = u + G_i_ + A_j_ + A_k_ + S_l_ + S_m_ + E_ijklm_ (1)

where Y_ijklm_ were the traits measured on each individual cow, μ was the overall population mean for the traits, G_i_ was the fixed effect associated with the genotype, A_i_ was the fixed effect due to the age, A_k_ was the fixed effect due to the age of dam, S_l_ was the fixed effect due to the season of sampling (spring *vs.* fall), S_m_ was the fixed effect due to the sire, and E_ijklm_ was the standard error.

We tested for an association between combined genotypes for different haplotypes with ultrasound carcass and body measurement traits, to explore any possible interaction between the different haplotypes. The following statistical linear model was used:

Y_ijklm_ = u + G_i_ + A_j_ + A_k_ + S_l_ + S_m_ + E_ijklm_ (2)

where Y_ijklm_, µ, A_j_, A_k_, S_l_, S_m_ and E_ijklm_ were the same as for model 1, and G_i_ was the fixed effect associated with combined genotype.

To avoid Type I errors derived from multiple statistical tests, we calculated the Bonferroni correction, which uses a modified criterion for significance (a/k, where *a* = 0.05, and k is the overall number of independent statistical tests conducted on the given data). In this study, we analyzed nine traits for two different SNPs, resulting in an adjusted *p*-value of 2.78 × 10^−3^ for the 5% significance threshold.

## 4. Conclusions

In summary, we showed that mRNA expression levels of the SIRT2 gene were highest in subcutaneous fat and kidney tissue of Qinchuan cattle. Two novel SNPs (g.19501 C >T and g.19518 C > T) in the 3'UTR of the gene were identified. Association analysis of the two SNPs with nine economic traits revealed that cattle with the C allele of g.19501 C > T and the T allele of g.19501 C > T loci had better body measurements. When in combination, the H_2_H_2_ diplotypes had better body size traits. Our findings suggest that the two SNPs could be used as molecular markers for eliminating or selecting preferred individuals in MAS breeding. This could facilitate the breeding of desired production traits in Qinchuan cattle.
